# Risk Factors of Thrombocytopenia After Cardiac Surgery with Cardiopulmonary Bypass

**DOI:** 10.21470/1678-9741-2021-0356

**Published:** 2023

**Authors:** Shujie Yan, Sizhe Gao, Song Lou, Qiaoni Zhang, Yuefu Wang, Bingyang Ji

**Affiliations:** 1 Department of Cardiopulmonary Bypass, Fuwai Hospital, Chinese Academy of Medical Science and Peking Union Medical College, Beijing, People’s Republic of China.; 2 Department of Anesthesiology, Fuwai Hospital, Chinese Academy of Medical Science and Peking Union Medical College, Beijing, People’s Republic of China.; 3 Department of Anesthesiology, Beijing Shijitan Hospital, Beijing, People’s Republic of China.

**Keywords:** Thrombocytopenia, Cardiopulmonary Bypass, Platelet Count, Body Surface Area, Blood Transfusion, Cardiac Surgery

## Abstract

**Introduction:**

Postoperative thrombocytopenia is common in cardiac surgery with cardiopulmonary bypass, and its risk factors are unclear.

**Methods:**

This retrospective study enrolled 3,175 adult patients undergoing valve surgeries with cardiopulmonary bypass from January 1, 2017 to December 30, 2018 in our institute. Postoperative thrombocytopenia was defined as the first postoperative platelet count below the 10^th^ quantile in all the enrolled patients. Outcomes between patients with and without postoperative thrombocytopenia were compared. The primary outcome was in-hospital mortality. Risk factors of postoperative thrombocytopenia were assessed by logistic regression analysis.

**Results:**

The 10^th^ quantile of all enrolled patients (75×10^9^/L) was defined as the threshold for postoperative thrombocytopenia. In-hospital mortality was comparable between thrombocytopenia and non-thrombocytopenia groups (0.9% *vs.* 0.6%, *P*=0.434). Patients in the thrombocytopenia group had higher rate of postoperative blood transfusion (5.9% *vs.* 3.2%, *P*=0.014), more chest drainage volume (735 [550-1080] *vs.* 560 [430-730] ml, *P*<0.001), and higher incidence of acute kidney injury (12.3% *vs.* 4.2%, *P*<0.001). Age > 60 years (odds ratio [OR] 2.25, 95% confidence interval [CI] 1.345-3.765, *P*=0.002], preoperative thrombocytopenia (OR 18.671, 95% CI 13.649-25.542, *P*<0.001), and cardiopulmonary bypass time (OR 1.088, 95% CI 1.059-1.117, *P*<0.001) were positively independently associated with postoperative thrombocytopenia. Body surface area (BSA) (OR 0.247, 95% CI 0.114-0.538, *P*<0.001) and isolated mitral valve surgery (OR 0.475, 95% CI 0.294-0.77) were negatively independently associated with postoperative thrombocytopenia.

**Conclusion:**

Positive predictors for thrombocytopenia after valve surgery included age > 60 years, small BSA, preoperative thrombocytopenia, and cardiopulmonary bypass time. BSA and isolated mitral valve surgery were negative predictors.

## INTRODUCTION

Postoperative thrombocytopenia is common in cardiac surgery with cardiopulmonary bypass (CPB), leading to increased postoperative blood loss. The etiology of thrombocytopenia in cardiac surgery is related to blood destruction, hemodilution, and platelet activation during CPB^[1]^.

The incidence of postoperative thrombocytopenia is about 10-40% according to different criteria of thrombocytopenia^[2,3,4]^. Postoperative thrombocytopenia was reported to be associated with postoperative adverse outcomes, including acute kidney injury (AKI), stroke, and death^[5,6,7]^. However, risk factors of postoperative thrombocytopenia are unclear. A study including 42 patients^[5]^ identifed preoperative platelet count, age, and intraoperative blood transfusion as independent predictors for postoperative thrombocytopenia. Given the small sample size, the conclusion was not convincing enough.

The present retrospective study was aimed to describe the distribution of postoperative thrombocytopenia in patients undergoing cardiac surgery with CPB, investigate the relationship between postoperative thrombocytopenia and outcomes, and identify risk factors of postoperative thrombocytopenia.

## METHODS

### Study Design and Population

This retrospective study was approved by our institutional review board (approval number 2020-1288), and the need for informed consent was waived. Data were extracted from the CPB database (2017-2018). Only valve surgeries were included to exclude the impact of perioperative antiplatelet drugs on platelet counts in coronary artery bypass grafting (CABG) and minimize surgical heterogeneity. The inclusion criteria were: (1) age > 18 years and (2) undergoing valve replacement or repair surgery from January 1, 2017 to December 30, 2018. The exclusion criteria included: (1) combined CABG, aortic surgery, or other surgery; (2) preoperative antiplatelet treatment; (3) previous cardiac surgery within six months; and (4) emergency surgery.

### Postoperative Thrombocytopenia

Platelet counts were extracted from blood routine tests at three time points: (1) last test before surgery (preoperative platelet count); (2) first test after surgery, immediately after admission to intensive care unit (ICU) (postoperative platelet count); and (3) last test before discharge from hospital (before-discharge platelet count).

Postoperative thrombocytopenia was defined as a postoperative platelet count below the 10^th^ quantile of all the enrolled patients, referring to Kertai MD’s method^[5]^. Platelet count recovery was defined as before-discharge platelet counts ≥125×109/L or the preoperative level.

### Risk Factors

Risk factors of postoperative thrombocytopenia were assessed. Variables contained demographic information, body surface area (BSA), comorbidity, European System for Cardiac Operative Risk Evaluation (EuroSCORE), preoperative laboratory tests, surgical information (including valve position, valve numbers, valve replacement or repair, and mechanical or bioprosthetic valve), CPB circuit, CPB time, aortic cross-clamping time, CPB temperature, and total heparin dose.

### Outcomes

Outcomes were compared between patients with and without postoperative thrombocytopenia. The primary outcome was in-hospital mortality. Secondary outcomes included chest drainage volume, postoperative blood transfusion, re-thoracotomy, platelet count recovery, cerebrovascular accidents (defined as the diagnosis of stroke or intracranial hemorrhage), AKI (defined as a postoperative increase in serum creatinine levels to more than two times baseline within a month or to > 354 mmol/l postoperatively), postoperative myocardial infarction (defined as an increase in creatine kinase to > 240 IU/l within 48 hours postoperatively), intraaortic balloon pump, and extracorporeal membrane oxygenation, mechanical ventilation time, and ICU length of stay. The postoperative red blood cell transfusion threshold was 8 g/L. Platelets were transfused when the level was < 50×10^9^/L. Fresh frozen plasma was transfused at excessive bleeding/chest drainage circumstances, with laboratory evidence suggesting coagulopathy.

### Surgical Procedure

Cardiac surgery was performed with mild hypothermic CPB (32-34ºC) under general anesthesia. CPB flow was maintained at 2.2-2.6 L/min/BSA (m^2^). A conventional or minimized CPB circuit was used according to the patient’s preoperative hemoglobin level and the perfusionists’ preference. A conventional CPB circuit consisted of a roller pump, a hollow-fiber oxygenator, an arterial flter, and an uncoated polyvinyl chloride circuit, with a priming volume of 1600 ml. A mini circuit^[8]^, with 900-ml priming volume, consisted of a hollow-fiber oxygenator integrated with arterial flter, smaller tubes, and vacuum-assisted venous reservoir drainage.

Unfractionated heparin (400 U/kg) was given to achieve activated clotting time (ACT) ≥ 410 seconds before CPB. ACT was maintained ≥ 410 seconds during CPB. After CPB was weaned of, protamine sulfate was administered for heparin reversal (1 mg protamine *vs.* 100 units heparin).

### Statistical Analysis

Variables were retrospectively collected from electric records. Continuous variables were presented as median (P25-P75) and were compared with the non-parametric test, according to its abnormal distribution. Categorical variables were presented as n (%) and compared with the x^2^ test for equal proportion. Logistic regression of multiple risk factors was performed to identify risk factors of postoperative thrombocytopenia. Univariable associations with *P*<0.10 and clinical significance were evaluated by a forward stepwise method to establish a logistic regression model of multiple risk factors. Nonlinear continuous variables were transformed into categorical variables, including age, preoperative platelet count, blood urea nitrogen (BUN), and international normalized ratio (INR). The variables with strong collinearity were chosen according to clinical significance. For example, prosthetic valve type (mechanical or bioprosthetic valve) was not put into the logistic regression analysis due to its strong correlation with age. The ft of the logistic regression analysis was evaluated by the Hosmer-Lemeshow test.

Two sensitivity analyses were also performed to determine if the risk factors of thrombocytopenia were afected by preoperative thrombocytopenia and intraoperative platelet transfusion. We excluded patients with preoperative platelet counts ≤ 125×109/L and patients who received intraoperative platelet transfusion, respectively. All statistical testing was two-sided, and a *P*-value < 0.05 was considered significant. All statistical analyses were performed using IBM Corp. Released 2015, IBM SPSS Statistics for Windows, Version 23.0, Armonk, NY: IBM Corp. The study was presented following the Strengthening the Reporting of Observational Studies in Epidemiology (or STROBE) guidelines.

## RESULTS

### Patients and Postoperative Platelet Count

**Fig. 1:**
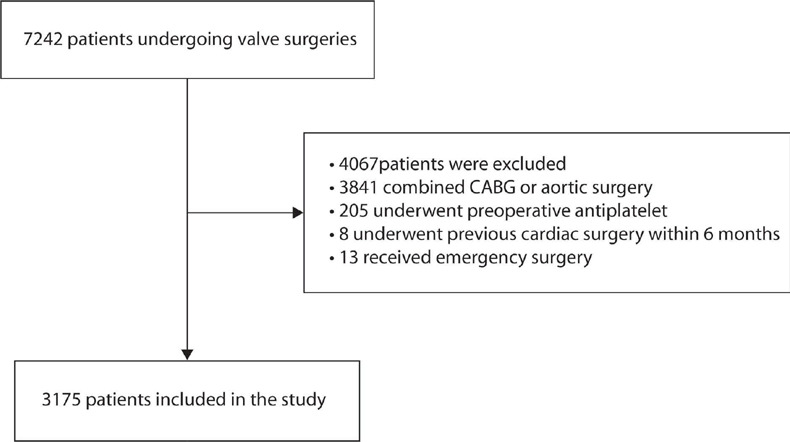
Flow diagram of patients’ inclusion and exclusion. CABG=coronary artery bypass grafting.

**Fig. 2:**
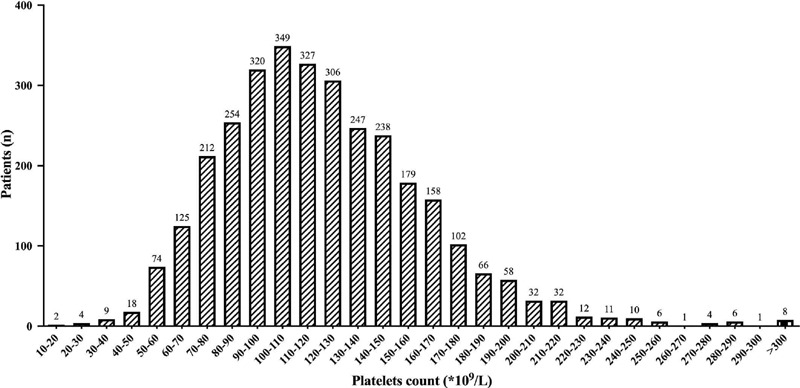
Distribution of postoperative platelet counts.

According to the inclusion and exclusion criteria, 3,175 patients were enrolled (Figure 1). The mean age was 54 years, and 1,735 (54.6%) were male. More than half of the patients’ first postoperative platelet counts were < 125×109/L (reference value in normal healthy individuals), 10% were < 75×109/L, and 1% were < 50×109/L. The 10th, 25th, 50th, and 75th quantile of postoperative platelet count were 75×109/L, 94×109/L, 117×109/L, and 146×109/L, respectively (Figure 2). The threshold for postoperative thrombocytopenia was defined as 75×109/L. The incidence of postoperative thrombocytopenia was 10.2% (n=324), and 85% of the patients had platelet recovery at hospital discharge.

### Pre and Intraoperative Information

Patients with postoperative thrombocytopenia were older (59.93 [51.92-65.94] *vs.* 54.15 [46.12-62.28] years, *P*<0.001), had lower body mass index (23 [21-25] *vs.* 24 [22-27] kg/m2, *P*<0.001), smaller BSA (1.64 [1.53-1.76] *vs.* 1.7 [1.58-1.85] m^2^, *P*<0.001), higher EuroSCORE (3 [2-4] *vs.* 2 [2-3], *P*<0.001), lower preoperative platelet count (131 [110-152] *vs.* 203 [170-240] × 109/L, *P*<0.001), lower preoperative hemoglobin level (136 [125-149] *vs.* 139 [127-150] g/L, *P*=0.04), higher BUN (6.6 [5.3-8.12] *vs.* 6.21 [5.1-7.6] mmol/L, *P*=0.003), and higher INR (1.07 [1.01-1.15] *vs.* 1.02 [0.98-1.08], *P*<0.001) level (Table 1).

**Table 1:** Preoperative data.

	No postoperative thrombocytopenia (n=2851)	Postoperative thrombocytopenia (n=324)	*P*-value
Age (years)	54.15 (46.12-62.28)	59.93 (51.92-65.94)	**< 0.001**
Male, n (%)	1558 (54.6%)	177 (54.6%)	0.995
BMI (kg/m^2^)	24 (22-27)	23 (21-25)	**< 0.001**
BSA (m^2^)	1.7 (1.58-1.85)	1.64 (1.53-1.76)	**< 0.001**
Comorbidity			
Diabetes, n (%)	184 (6.5%)	24 (7.4%)	0.511
Hypertension, n (%)	823 (28.9%)	83 (25.6%)	0.22
Hyperlipidemia, n (%)	1066 (37.4%)	116 (35.8%)	0.575
Chronic kidney disease, n (%)	8 (0.3%)	1 (0.3%)	1
Smoker, n (%)	868 (30.4%)	100 (30.9%)	0.877
EuroSCORE	2 (2-3)	3 (2-4)	**< 0.001**
Preoperative LVEF (%)	60 (58-65)	60 (58-65)	0.413
Preoperative laboratory test			
Platelet count (×10^9^/L)	203 (170-240)	131 (110-152)	**< 0.001**
Hemoglobin (g/L)	139 (127-150)	136 (125-149)	**0.04**
Creatinine (µmol/L)	80.47 (70-93)	81.48 (70.55-94.67)	0.215
Blood urea nitrogen (mmol/L)	6.21 (5.1-7.6)	6.6 (5.3-8.12)	0.003
Alanine transaminase (U/L)	18 (13-28)	18 (13-26)	0.747
Albumin (g/L)	41.8 (39.2-44.6)	41.5 (38.65-44.2)	0.161
PT-INR	1.02 (0.98-1.08)	1.07 (1.01-1.15)	**< 0.001**
Preoperative RBC transfusion (U)	0 (0-0)	0 (0-0)	0.061
Preoperative anticoagulants, n (%)	1205 (42.3%)	158 (48.8%)	**0.025**

Data are presented as n (%) or median (interquartile range). BMI=body mass index; BSA=body surface area; EuroSCORE=European System for Cardiac Operative Risk Evaluation; LVEF=left ventricular ejection fraction; PT-INR=prothrombin time-international normalized ratio; RBC=red blood cell

In regard to valve surgical position and type, more patients in the postoperative thrombocytopenia group underwent multivalve surgery (59.9% *vs.* 43.6%, *P*<0.001) and bioprosthetic valve replacement surgery (32.7% *vs.* 19.2%, *P*<0.001). More patients in the non-thrombocytopenia group underwent isolated mitral valve surgery (21% *vs.* 7.1%, *P*<0.001) and valve repair surgery (18.8% *vs.* 9%, *P*<0.001). In addition, postoperative thrombocytopenia was associated with longer CPB (114 [86-147] *vs.* 93 [74-121] minutes, *P*<0.001) and aortic cross-clamping times (85 [61-110] *vs.* 67 [52-89] minutes, *P*<0.001), lower nadir temperature (31.8 [30.9-32.6] *vs.* 32.1 [31.3-32.7] ºC, *P*<0.001), lower heparin dosage (30000 [26800-34400] *vs.* 32400 [28400-36800] IU, *P*<0.001), and more allogeneic blood transfusion during the surgery (Table 2).

**Table 2:** Intraoperative data.

	No postoperative thrombocytopenia (n=2851)	Postoperative thrombocytopenia (n=324)	*P*-value
Surgical procedure			
Valve position			**< 0.001**
Mitral valve, n (%)	598 (21%)	23 (7.1%)	**< 0.001**
Tricuspid valve, n (%)	63 (2.2%)	7 (2.2%)	0.954
Aortic valve, n (%)	948 (33.3%)	100 (30.9%)	0.387
Mitral and tricuspid valves, n (%)	751 (26.3%)	94 (29%)	0.303
Mitral and aortic valves, n (%)	179 (6.3%)	31 (9.6%)	**0.024**
Aortic and tricuspid valves, n (%)	19 (0.7%)	4 (1.2%)	0.253
Mitral, aortic, and tricuspid valves, n (%)	293 (10.3%)	65 (20.1%)	**< 0.001**
Valve type*			**< 0.001**
Valve repair, n (%)	536 (18.8%)	29 (9%)	**< 0.001**
Valve replacement (mechanical valve), n (%)	1768 (62%)	189 (58.3%)	0.197
Valve replacement (bioprosthetic valve), n (%)	547 (19.2%)	106 (32.7%)	**< 0.001**
Multivalve, n (%)	1242 (43.6%)	194 (59.9%)	**< 0.001**
Thoracoscopic procedure, n (%)	19 (0.7%)	0 (0%)	0.25
Valvular vegetation, n (%)	16 (0.6%)	2 (0.6%)	0.705
Perivalvular leakage, n (%)	6 (0.2%)	2 (0.6%)	0.193
Cardiopulmonary circuits			0.952
Conventional circuit, n (%)	2607 (92.6%)	295 (92.5%)	
Mini circuit with VAVD, n (%)	178 (6.3%)	21 (6.6%)	
Mini circuit without VAVD, n (%)	31 (1.1%)	3 (0.9%)	
Cardiopulmonary bypass time (minutes)	93 (74-121)	114 (86-147)	**< 0.001**
Aortic cross-clamping time (minutes)	67 (52-89)	85 (61-110)	**< 0.001**
Nadir nasal temperature (ºC)	32.1 (31.3-32.7)	31.8 (30.9-32.6)	**< 0.001**
Nadir bladder temperature (ºC)	33 (32.1-33.7)	32.5 (31.5-33.3)	**< 0.001**
Ultrafltration volume (ml)	1000 (0-1500)	1000 (0-2000)	**0.001**
Heparan dose (U)	32400 (28400-36800)	30000 (26800-34400)	**< 0.001**
Intraoperative RBC transfusion (U)	0 (0-0)	0 (0-4)	**< 0.001**
Intraoperative FFP transfusion (ml)	0 (0-0)	0 (0-400)	**< 0.001**
Intraoperative platelet transfusion (U)	0 (0-0)	0 (0-0)	**< 0.001**

"Data are presented as n (%) or median (interquartile range). *Valve type, surgeries with more valves.

FFP=fresh frozen plasma; RBC=red blood cell; VAVD=vacuum-assisted venous drainage

### Outcomes

Outcomes were compared between patients with and without postoperative thrombocytopenia. In-hospital mortality was comparable between non- thrombocytopenia and thrombocytopenia groups (0.6% *vs.* 0.9%, *P*=0.343). Patients with postoperative thrombocytopenia had more chest drainage volume (735 [550-1080] *vs.* 560 [430-730] ml, *P*<0.001), higher rates of postoperative blood transfusion (5.9% *vs.* 3.2%, *P*=0.014), and lower frequency of platelet recovery at hospital discharge (66.2% *vs.* 88.5%, *P*<0.001). In addition, postoperative thrombocytopenia was associated with adverse outcomes including AKI (12.3% *vs.* 4.2%, *P*<0.001), longer mechanical ventilation (19 [15-35] *vs.* 16 [12-20] hours, *P*<0.001), and longer ICU length of stay (3 [2-4] *vs.* 2 [1-4] days, *P*=0.001) (Table 3).

**Table 3:** Postoperative outcomes.

	No postoperative thrombocytopenia (n=2851)	Postoperative thrombocytopenia (n=324)	*P*-value
Primary outcome			
In-hospital mortality, n (%)	16 (0.6%)	3 (0.9%)	0.434
Secondary outcome			
Platelet recovery, n (%)	2475 (88.5%)	208 (66.2%)	**< 0.001**
Chest drainage volume (ml)	560 (430-730)	735 (550-1080)	**< 0.001**
Postoperative transfusion, n (%)	92 (3.2%)	19 (5.9%)	**0.014**
RBC, n (%)	80 (2.8%)	18 (5.6%)	**0.007**
FFP, n (%)	41 (1.4%)	11 (3.4%)	**0.009**
Platelet, n (%)	15 (0.5%)	6 (1.5%)	**0.043**
Re-thoracotomy, n (%)	47 (1.6%)	10 (3.1%)	0.065
Stroke, n (%)	11 (0.4%)	3 (0.9%)	0.165
Myocardial infarction, n (%)	4 (0.1%)	1 (0.3%)	0.416
Hepatic failure, n (%)	19 (0.7%)	9 (2.8%)	**0.001**
Acute kidney injury, n (%)	121 (4.2%)	40 (12.3%)	**< 0.001**
CRRT, n (%)	13 (0.5%)	3 (0.9%)	0.219
IABP, n (%)	9 (0.3%)	2 (0.6%)	0.311
ECMO, n (%)	3 (0.1%)	0 (0%)	1
Prolonged mechanical ventilation, n (%)	140 (4.9%)	46 (14.2%)	**< 0.001**
ICU length of stay (days)	2 (1-4)	3 (2-4)	**0.001**
Mechanical ventilation time (hours)	16 (12-20)	19 (15-35)	**< 0.001**

Data are presented as n (%) or median (interquartile range). CRRT=continuous renal replacement therapy; ECMO=extracorporeal membrane oxygenation; FFP=fresh frozen plasma; IABP=intra-aortic balloon pump; ICU=intensive care unit; RBC=red blood cell

### Risk Factors of Postoperative Thrombocytopenia

Logistic regression of multiple risk factors was performed to identify risk factors of postoperative thrombocytopenia. Fifteen covariables were selected: age, BSA, EuroSCORE, preoperative platelet count, BUN, INR, preoperative blood transfusion, preoperative anticoagulant use, CPB time, valve position, valve repair/replacement, nasal nadir temperature, intraoperative ultrafltration volume, and intraoperative heparin dose. According to the logistic regression analysis, age > 60 years (odds ratio [OR] 2.25, 95% confidence interval [CI] 1.345-3.765, *P*=0.002), preoperative thrombocytopenia (OR 18.671, 95% CI 13.649-25.542, *P*<0.001), and CPB time (OR 1.088, 95% CI 1.059-1.117, *P*<0.001) were positive predictors for postoperative thrombocytopenia (Table 4). BSA (OR 0.247, 95% CI 0.114-0.538) and isolated mitral valve surgery (OR 0.475, 95% CI 0.294-0.77) were negative predictors for postoperative thrombocytopenia. These five risk factors were confirmed by sensitivity analyses excluding patients with preoperative platelet counts ≤ 125×109/L and patients who received intraoperative platelet transfusion (Table 5).

**Table 4:** Multivariable analysis for postoperative thrombocytopenia*.

	OR (95% CI)	*P*-value
Age (years)		
18-40	Reference category	
40-60	1.336 (0.798-2.238)	0.27
> 60	2.25 (1.345-3.765)	0.002
Body surface area	0.247 (0.114-0.538)	< 0.001
Cardiopulmonary bypass time (per 10-minute increase)	1.088 (1.059-1.117)	< 0.001
Isolated mitral valve	0.475 (0.294-0.77)	0.002
Preoperative thrombocytopenia	18.671 (13.649-25.542)	< 0.001

*The final multivariable logistic analysis was based on n=2910 due to missing data of 265 subjects. Predictive ability of the model was 91.1%. *P*-value of Hosmer-Lemeshow test was 0.469. CI=confidence interval; OR=odds ratio

**Table 5:** Sensitivity analysis of multivariable analysis for postoperative thrombocytopenia.

	OR (95% CI)	*P*-value
*Patients with intraoperative platelet transfusion excluded* *		
Age (years)		
18-40	Reference category	
40-60	1.251 (0.727-2.153)	0.419
> 60	2.049 (1.191-3.525)	0.01
Body surface area	0.213 (0.093-0.488)	< 0.001
Cardiopulmonary bypass time (per 10-minute increase)	1.086 (1.053-1.121)	< 0.001
Isolated mitral valve	0.431 (0.256-0.723)	0.001
Preoperative thrombocytopenia	21.959 (15.541-31.028)	< 0.001
*Patients with preoperative thrombocytopenia excluded#*		
Age (years)		
18-40	Reference category	
40-60	1.333 (0.717-2.477)	0.364
> 60	2.864 (1.561-5.254)	0.001
Body surface area	0.172 (0.068-0.439)	< 0.001
Cardiopulmonary bypass time (per 10-minute increase)	1.1 (1.067-1.134)	< 0.001
Isolated mitral valve	0.575 (0.341-0.968)	0.037

*The final multivariable logistic analysis was based on n=2748 due to missing data of 254 subjects. Predictive ability of the model was 92%. *P*-value of Hosmer-Lemeshow test was 0.607. ^#^The final multivariable logistic analysis was based on n=2668 due to missing data of 217 subjects. Predictive ability of the model was 94.2%. *P*-value of Hosmer-Lemeshow test was 0.552. CI=confidence interval; OR=odds ratio

## DISCUSSION

This retrospective study gave an overview of postoperative thrombocytopenia after cardiac surgery. In-hospital mortality was not increased in the thrombocytopenia group. Patients with postoperative thrombocytopenia tended to have more postoperative bleeding and required more blood transfusion. In addition, postoperative thrombocytopenia was accompanied by a series of complications, including AKI. Predictors for postoperative thrombocytopenia included age > 60 years, BSA, preoperative thrombocytopenia, CPB time, and isolated mitral valve surgery.

There is no universally accepted definition of postoperative thrombocytopenia in cardiac surgery. Platelet count decline was typical in patients after cardiac surgery with CPB, and many patients’ platelet counts were below the reference value in normal healthy individuals. But the threshold of platelet count with the most crucial clinical significance was undetermined. The definition of postoperative thrombocytopenia in the present study was < 75×10^9^/L, which was the 10^th^ quantile of the study population. Two previous large studies also took 75×109 as the threshold of postoperative thrombocytopenia^[5,6]^. They reported that postoperative platelet count < 75×10^9^ was associated with adverse outcomes, including mortality, AKI, and prolonged ICU length of stay^[5,6]^.

The present study did not find an association between postoperative thrombocytopenia and in-hospital mortality. But our study demonstrated that patients with postoperative thrombocytopenia had a higher incidence of AKI and longer mechanical ventilation time and ICU length of stay, which was consistent with the abovementioned two studies. We also found the relationship between postoperative thrombocytopenia with transfusion and bleeding. Previous studies reported similar results^[9]^.

The mechanism under the relationship between thrombocytopenia and adverse outcomes is unclear. Thrombocytopenia has also been identifed as a risk factor of multi-organ failure in critically ill patients^[10,11,12,13]^. Postoperative thrombocytopenia might be coupled with massive platelet-derived chemokines, which were associated with multi-organ injury^[14,15]^. Besides, postoperative thrombocytopenia and other adverse outcomes might be both the consequences of prolonged CPB and some underlying situations (*i.e.*, microvascular thrombosis^[16]^). Another possible assumption was that postoperative thrombocytopenia led to more bleeding and transfusions, which increased the risks of AKI and other adverse outcome^[17,18]^.

Platelet consumption and hemodilution were considered as the leading causes of platelet count decline after cardiac surgery with CPB. Platelet was an essential modulator in infammation and hemostasis. Once CPB was started, platelets were activated by mechanical shear stress and artificial surface contact, and platelet activation cascade was induced^[1]^. High shear stress could also induce platelet apoptosis^[19]^. In addition, hemolysis during CPB enhanced platelet aggregation^[20]^. Our study identifed age > 60 years, BSA, preoperative thrombocytopenia, and CPB time as independent risk factors of postoperative thrombocytopenia. These factors were related to platelet consumption and hemodilution. Patients with small BSA had more hemodilution during CPB. Prolonged CPB indicated more platelet activation. Older age was reported to be associated with platelet hyperreactivity and enhanced aggregation^[21]^. Therefore, we guessed older patients might have more platelet activation during cardiac surgery. In addition, older individuals had fewer platelet counts than younger people^[22]^.

In addition, the present study found a relationship between valve surgical information and postoperative thrombocytopenia. Isolated mitral valve surgery was a negative predictor for thrombocytopenia. The reasons might be stated as follows. Firstly, compared to multivalve surgeries, isolated mitral valve surgeries usually required shorter CPB time and therefore caused less platelet consumption. Secondly, compared to isolated aortic valve surgeries, a larger proportion of isolated mitral valve surgeries was valve repair surgery. Prosthetic valves would lead to increased platelet activation^[23]^.

For patients with high risks of postoperative thrombocytopenia, an active platelet preservation strategy might be applied. Heparinbonded cardiopulmonary circuit reduced platelet activation compared to standard uncoated circuit^[24]^. Several potential agents have been reported, including nitric oxide^[25]^. More research is required to obtain comprehensive recognition of platelet preservation during CPB.

Heparin-induced thrombocytopenia (HIT) was also a big concern in postoperative thrombocytopenia. A biphasic platelet count pattern was typical of HIT, while early-onset thrombocytopenia was mainly related to CPB^[26]^. In the present study, postoperative thrombocytopenia was defined with the first platelet count test after surgery, and hence HIT was not the primary concern. We screened all the patients with postoperative thrombocytopenia and those who received postoperative platelet transfusion retrospectively. Only three patients were suspected with HIT, but not confirmed as anti-PF4/heparin antibody test was not available in our institute.

### Limitations

This study had several limitations, mainly due to its retrospective nature. Firstly, postoperative platelet count was affected by intraoperative platelet transfusion. However, a sensitivity analysis excluding patients with intraoperative platelet transfusion was performed and obtained similar results. Secondly, the result might be influenced by the definition of postoperative thrombocytopenia. Thirdly, platelet function and markers of platelet activation were not tested. Fourthly, other factors (*i.e.*, redo surgery and valve brands), which might be associated with postoperative thrombocytopenia, were not included in risk factor analysis.

## CONCLUSION

Thrombocytopenia was common after cardiac surgery. The risk factors of postoperative thrombocytopenia included age > 60 years, BSA, preoperative thrombocytopenia, and CPB time. Patients with postoperative thrombocytopenia tended to have more postoperative bleeding and required more blood transfusion, and post thrombocytopenia was associated with a series of complications.
